# Valorization of wood waste for enhanced polyhydroxybutyrate production by *Klebsiella* sp. MK3

**DOI:** 10.1038/s41598-025-01305-7

**Published:** 2025-05-25

**Authors:** Mukesh Kumar, Rohan Samir Kumar Sachan, Arun Karnwal, Dharmesh Sur, Rahul Thakur, Abhinav Kumar, Sathiyamoorthy Manickkam, Abinet Gosaye Ayanie, Hamad Almujibah

**Affiliations:** 1 Department of Microbiology, School of Bioengineering and Biosciences, Lovely Professional University, Phagwara, 144411 Punjab India; 2 Department of Medical Laboratory Sciences, GNA University, Phagwara, 144401 Punjab India; 3 Department of Microbiology, Graphic Era (Deemed to be University), Dehradun, Uttarakhand India; 4 Department of Chemical Engineering, Faculty of Engineering & Technology, Marwadi University Research Center, Marwadi University, Rajkot, 360003 Gujarat India; 5 Department of Chemistry, Chandigarh Engineering College, Chandigarh Group of Colleges-Jhanjeri, Mohali, 140307 Punjab India; 6 Department of Nuclear and Renewable Energy, Ural Federal University Named after the First President of Russia Boris Yeltsin, Ekaterinburg, 620002 Russia; 7 Faculty-Chemical Engineering, Higher Colleges of Technology, Ruwais Women’s College, Abu Dhabi, United Arab Emirates; 8 Department of Mechanical Engineering, Adama Science and Technology University, Adama, 2552 Ethiopia; 9 Centre for Research Impact & Outcome, Chitkara University Institute of Engineering and Technology, Chitkara University, Rajpura, India; 10 Department of Mechanical Engineering and Renewable Energy, Technical Engineering College, The Islamic University, Najaf, Iraq; 11 Department of Civil Engineering, College of Engineering, Taif University, P.O. Box 11099, Taif City, 21974 Saudi Arabia

**Keywords:** Polyhydroxybutyrate (PHB), Wood waste valorization, *Klebsiella* sp. MK3, Response surface methodology (RSM), Plackett-Burman design (PBD), Statistical experimental design., Microbiology, Chemical engineering

## Abstract

The valorization of wood waste as a sustainable bacterial feedstock for the production of Polyhydroxybutyrate (PHB) is explored in this study, aiming to provide an environmentally friendly alternative to conventional plastics. Wood waste, treated with 4% sulfuric acid, served as the carbon source for isolating bacteria from Jalandhar waste streams, with the strain *Klebsiella* sp. MK3 identified as the most effective in PHB production after 16s rRNA sequencing. Analytical methods including the Molisch test, DNS, and sugar utilization tests confirmed sugar presence and consumption by the bacterial isolate. Media optimization using Design Expert 12.0 utilized a quadratic model, achieving a robust fit with an R² value of 98.6%. Optimization via Plackett-Burman design and response surface methodology enhanced PHB yield to 4.37 mg/mL, a significant increase over previous benchmarks. This yield was achieved under optimal conditions of 1.7% carbon concentration, 0.105% nitrogen concentration, and a constant temperature of 37 °C. Qualitative analysis of PHB by UV-Vis spectroscopy, FTIR, and NMR confirmed its purity and composition. The study highlights the potential of wood waste and wastewater as substrates for cost-effective PHB production, with significant applications in packaging, agriculture, medicine, and more, thus promoting reduced reliance on non-renewable resources and advancing sustainability goals.

## Introduction

Plastic pollution represents a severe environmental issue due to its persistent, non-degradable nature in the environment^1^. The extensive use of traditional plastics, derived from non-renewable petroleum resources, has led to widespread environmental degradation, contributing significantly to landfills and oceanic waste. The durability that makes plastics so useful also renders them a long-lasting pollutant, affecting wildlife and ecosystems globally^1,2^.

Traditional plastics, derived from fossil fuels, have contributed significantly to environmental pollution due to their non-biodegradable nature, resulting in microplastic accumulation and ecosystem disruption^3,4^. Polyhydroxybutyrate (PHB), a microbial biopolymer, offers a sustainable alternative, being fully biodegradable and biocompatible. Produced by strains like *Cupriavidus necator* and *Bacillus megaterium* under nutrient-limited conditions, PHB exhibits thermoplastic properties comparable to polypropylene^5,6^. It degrades naturally in the environment through microbial action, transforming into water, carbon dioxide, and biomass under aerobic conditions, and methane under anaerobic conditions^4^. Significant microbial strains capable of PHB production include *Bacillus* spp., *Cupriavidus necator*, and certain genetically engineered strains of E. coli, which are known for their efficiency in PHB synthesis^5^. PHB is not only comparable to conventional plastics in terms of physical properties such as thermoplasticity and biocompatibility but also features material characteristics that are favorable for medical applications, packaging, agricultural films, and even as a feedstock for 3D printing^6^.

Prior studies have demonstrated the effective utilization of wood waste as a substrate for the production of polyhydroxyalkanoates (PHAs), emphasizing its potential to enhance yield while promoting sustainability. For instance, the study by Li et al. (2021) highlighted the conversion of wood hydrolysates into PHAs using microbial fermentation. The hydrolysates provided essential carbon sources such as glucose and xylose, enhancing PHA production without requiring expensive purification steps. Additionally, wood waste utilization addresses dual objectives: reducing environmental waste and providing an economical alternative to refined substrates. Optimized hydrolysis conditions have been shown to increase sugar release, directly correlating to improved microbial growth and PHA accumulation^7,8^. However, research on using wood waste is less extensive despite its rich carbon content, predominantly composed of cellulose and hemicellulose, which can be hydrolyzed into fermentable sugars using acid treatments like sulfuric acid. This process enhances the accessibility of carbon, improving the substrate’s fermentability and, subsequently, the yield of PHB. Previous study demonstrated a significant enhancement in PHB yield when utilizing treated wood waste, underscoring its potential as an effective feedstock^7^. In India, wood like Sal and tea wood are commonly used in timber mills for furniture and various purposes. The ready availability of this wood makes it a suitable choice as feedstock for the proposed research.

This research aims to further explore the valorization of wood waste for PHB production by optimizing bioprocess conditions through factorial design and Response Surface Methodology (RSM). It seeks to address the challenges associated with the low fermentability of lignocellulosic materials and to achieve an efficient, cost-effective, and environmentally friendly production process. The novelty of this study lies in its approach to integrating wood waste valorization with microbial synthesis of PHB, aiming to contribute substantially to sustainable waste management and plastic production.

## Materials and methods

### Sample collection, isolation, and screening of PHB producing bacteria

Sewage water samples were collected using grab sampling technique from four locations of Jalandhar, Punjab (Sample 1 (L-1) location: latitude 31° 16.1126’ N, longitude 75° 35.7164’ E, Sample 2 (L-2) location: latitude 31° 20.2469’ N, and longitude 75° 40.1239 E, Sample 3 (L-3) location: latitude 31° 20.4499’ N, and longitude 75° 31.7189’ E, Sample 4 (L-4) location: latitude 31° 13.6414’ N, and longitude 75° 38.2179 E)^9^. All the sample collected were inoculated into sterile nutrient broth and labeled as mother samples and subjected to serial dilution, and aliquot of 0.1 mL was spread on MSM agar along with 10 g/L sucrose, 0.225 mg/L Nile blue, and 15 g/L agar. Incubation at 30 °C for 48 h facilitated primary screening, where Nile blue incorporated into bacterial cytoplasm during growth. Under U.V. light, plates exhibited fluorescence, allowing identification of fluorescent colonies as potential PHB producers^10^. Sudan Black staining was conducted on heat-fixed samples using a solution made by dissolving 0.3 g of Sudan Black B in 75 mL of 95% ethanol, then diluting it to 100 mL with distilled water. The samples were stained with this solution for 10 min, dried using filter paper, and clarified with drops of xylene. After another round of drying, they were counterstained with a 0.5% aqueous safranine solution for 5 s. Blue black colonies were observed and picked as confirmation of PHB producers^11^.

### PHA production, extraction, and identification

#### Production of PHB using MSM media

The isolates were inoculated in test tubes containing 10 mL of mineral salts medium (MSM) for PHB production. The inoculated tubes were incubated at 37 °C and 150 rpm for a duration of 72 h. The MSM composition for PHB production included ammonium chloride (1.5 g/L), yeast extract (0.16 g/L), KH_2_PO_4_ (1.52 g/L), Na_2_HPO_4_ (4.0 g/L), MgSO_4_·7H_2_O (0.52 g/L), CaCl_2_ (0.02 g/L), glucose (20 g/L), and a trace element solution (0.1 mL). The trace element solution contained ZnSO_4_·7H_2_O (0.13 g/L), FeSO_4_·7H_2_O (0.02 g/L), (NH_4_)_6_MO_7_O_24_. 4H_2_O (0.06 g/L), and H_3_BO_3_ (0.06 g/L). Glucose and trace element solutions were autoclaved separately and reconstituted before inoculation^12,13^.

#### Extraction process of PHB using hypochlorite method

Following incubation at 37 °C for 72 h, the production media was centrifuged at 12,000 rpm for 15 min. Pellets obtained were treated with 13% sodium hypochlorite for cell digestion, followed by incubation at 50 °C for 2 h. The resulting cell extract was centrifuged at 8000 RPM for 15–20 min, followed by separate washes with distilled water, acetone, and methanol. The residual white-colored precipitates were identified as PHB granules^14^. The heated chloroform was carefully placed in a sterile petri plate or watch glass, with PHB granules as chloroform has ability to dissolve PHB. After some hours of incubation at room temperature, chloroform eventually evaporates and a thin, light-colored PHB film became visible in the Petri plate^15^.

The dry biomass of the extracted PHB was quantified in g/L. The residual biomass was determined as the difference between the dry cell biomass and the dry biomass of PHB. The percentage of intracellular PHB accumulation was computed as the proportion of PHB in the dry cell weight. (DCW = Total dry cell weight)^16^.

$${\text{The dry weight of extracted PHB }}\left( {{\mathrm{g}}/{\mathrm{mL}}} \right)\,=\,{\text{DCW }}\left( {{\mathrm{g}}/{\mathrm{mL}}} \right)-{\text{Residual biomass }}\left( {{\mathrm{g}}/{\mathrm{mL}}} \right)$$

$${\text{PHB accumulation }}\left( \% \right)\,=\,{\text{Dry weight of extracted PHB }}\left( {{\mathrm{g}}/{\mathrm{mL}}} \right) \times {\mathrm{1}}00{\text{ }}/{\text{ DCW }}\left( {{\mathrm{g}}/{\mathrm{mL}}} \right)$$

#### Genotypic characterization Pf the potent strain by 16s rRNA sequencing

The PCR process began with the extraction and purification of DNA from an overnight bacterial culture using a series of steps involving centrifugation and enzymatic treatments. Universal primers (U5F and U4R) targeting conserved regions of the 16 S rRNA gene were employed for PCR amplification, ensuring amplification from any bacterial species. Each PCR reaction contained 100 ng of purified DNA in a 20 µl reaction mixture consisting of 10X Buffer, deoxy-nucleoside tri-phosphates, primers, and Taq polymerase. PCR cycles included initial denaturation at 95 °C for 3 min, followed by 32 cycles of denaturation at 95 °C for 30 s, annealing at 55 °C for 30 s, and extension at 72 °C for 1 min. A final extension cycle at 72 °C for 10 min concluded the amplification. Analysis of the PCR products was performed by electrophoresis on 1.5% agarose gel containing ethidium bromide, followed by visualization under UV illumination. This comprehensive PCR protocol facilitated the amplification of a segment (~ 1200 bp) of the 16 S rRNA gene, enabling the identification and characterization of bacterial species present in the initial culture^17^.

### Hydrolysis of wood waste

Sal and teak wood waste obtained from Phagwara and Jalandhar was subjected to collection and sieving. Subsequently, 10% w/v of the wood waste was blended with waste-water water (100 mL) and 4% v/v sulphuric acid. The resulting mixture was incubated at 120 degrees for 1 h. Following incubation, the mixture was filtered using muslin cloth, separating the supernatant. Quantitative and qualitative assessments, including the Molisch test, DNS spectrophotometry, and sugar utilization test, were then conducted on the supernatant to determine sugar concentration after adjusting pH at 7^12,13^.

### Optimization using statistical experimental design

The utilization of Design of Experiments (DoE) or Statistical Experimental Design (SED), particularly through the Plackett and Burman (P.B.) orthogonal arrays, offers an efficient method for screening influential factors in PHB production. By systematically organizing experiments and minimizing the number of variables tested, this approach provides accurate estimations of direct effects. Employing PBD, the fundamental values of independent variables were coded into High (+ 1) and Low (– 1) levels, and the resulted response was analysed relatively at 95% significance level. Overall, this strategy aids in selecting relevant factors, defining appropriate ranges, and preventing experimental failures, thus facilitating the development of sustainable PHB production processes^18^.

**Table 1:** Illustrating higher and lower values of factors used in PBD.

Sr. no.	Factors	Low level	Higher level
1.	Carbon	1%	4%
2.	Nitrogen	0.01%	0.2%
3.	Ferric citrate	0.001%	0.01%
4.	Temperature	28	40
5.	pH	5	9
6.	Inoculum size	2	10
7.	MgSO_4_.7H_2_O	0.01	0.1
8.	Na_2_HPO_4_	0.1	0.4
9.	K_2_HPO_4_	0.1	0.4
10.	Incubation period	48	96
11.	Trace elements	0.1	1

Biopolymer synthesis was conducted in a 100 mL Erlenmeyer flask with a 50 mL working volume for the minimal medium. The formulation of the production media, as shown in Table 1, about parameters such as carbon and nitrogen concentrations, pH, temperature, ferric citrate, incubation period, inoculum size, KH_2_PO_4_, Na_2_HPO_4_, MgSO_4_.7H_2_O, and trace elements, was determined utilizing Design Expert 12.0 software. To ensure reproducibility, each step of the experiment in the flask was carried out in triplicates. Further optimization, considering the most influential factors, was conducted using Response Surface Methodology (RSM)^19^. RSM, coupled with a well-designed experiment, has become a prevalent approach for formulation optimization. Carbon concentration (1–4%), nitrogen concentration (0.01–0.2%), and temperature (28 °C to 40 °C) were identified as the three primary factors significantly affecting the production rate of PHB. 6 center points,2 axil (star) point replications were accounted for this design. DoE of these factors is as mentioned in the Table 1.

### Qualitative analysis of PHB using UV-Vis spectroscopy

The granules or PHB films were blended in 10 mL of concentrated sulfuric acid and subjected to a water bath for 15 min at 100 °C. This process transformed the polymer into brown-colored crotonic acid, which was subsequently cooled and analyzed using a UV spectrophotometer at 235 nm, comparing it to the sulfuric acid blank. The UV spectrophotometer quantified the PHB content in the extracted samples at 235 nm, referencing the standard graph of 3-hydroxybutyric acid^20,21^.

### Fourier transform-infrared spectroscopy (FT-IR analysis)

The PHB extracted from the organism was analysed by Fourier-transform infrared spectroscopy (FT-IR) analysis using JASCO FT/IR instrumentation. The analysis was conducted within a spectral range of 4000 –400 cm⁻¹ to validate the functional groups present in the extracted polymer^22^.

### 1H and 13C NMR

To conduct carbon-13 (C-NMR) analysis, we followed the method outlined by^23^ This involves preparing a solution of PHB by dissolving it in a deuterated solvent, typically deuterated chloroform, and then transferring it into an NMR tube. The spectrum is then obtained using an appropriate NMR spectrometer, and chemical shifts are compared to the solvent peak. Proton (1H-NMR) analysis follows a similar process, where the sample is dissolved in a deuterated solvent like deuterated chloroform. Both 13C and 1H-NMR spectra provide valuable insights into the polymer’s structure, with 13C-NMR revealing carbon connections and 1H-NMR offering information on hydrogen environments.

## Results and discussion

### Isolation of PHB producer

Following the Nile blue staining isolation process, 26 isolates were identified on agar plates, labelled as samples 1 to 26 or S-1 to S-26. Subsequent Sudan black staining verified 19 isolates, all of which exhibited positive indications of being potential PHB producers, as detailed in Table 2.

**Table 2:** Sudan black staining of different selected isolates.

Isolates no.	Observation	Isolates no.	Observations
S-1	Positive	S-14	Positive
S-2	Positive	S-15	Negative
S-3	Positive	S-16	Positive
S-4	Positive	S-17	Negative
S-5	Positive	S-18	Positive
S-6	Negative	S-19	Positive
S-7	Positive	S-20	Positive
S-8	Negative	S-21	Positive
S-9	Positive	S-22	Negative
S-10	Negative	S-23	Negative
S-11	Positive	S-24	Positive
S-12	Positive	S-25	Positive
S-13	Positive	S-26	Positive

### Extraction and quantification of PHB

After the extraction process and washing, a white precipitate remained, which was estimated as the pure PHB content. It was measured for further study of the production rate of PHB as shown in Table 3.

**Table 3:** Showing DCW of PHB and its production percentage.

Sample name	DCW (mg/mL) (Mean)	Dry weight of extracted PHA (mg/mL) (mean)	Expected % of the PHA (mean)
1	10.45	6.05	57
2	10.35	5.9	56.5
3	10.05	6.75	67
4	9.75	4.25	41.9
5	9.85	6.05	60.6
7	10.2	4.05	38
9	9.9	3.05	28.8
11	9.95	2.25	21.8
12	10.15	5.45	52.7
13	9.4	5.65	60.2
14	9.6	4.05	42.1
16	10.1	5.45	53.6
18	9.65	4.25	43
19	9.9	3.25	31.5
20	10.4	3.5	31.4
21	10.3	2.75	26.6
24	9.7	4.05	40.8
25	9.85	3	28.8
26	10.65	4.25	39.6

After overnight incubation of chloroform and PHB mixture, chloroform evaporates and a distinct, light white-coloured, thin layer of PHB film became visible in the Petri plate, upon meticulous examination of the DCW, it was evident that isolate no. 3 exhibited the highest production of PHB. Consequently, isolate no. 3 was identified as the optimal strain for subsequent production, utilizing wood waste as the substrate medium, and subjected to statistical analysis. To identify the isolate, 16 S sequencing was carried out.

### 16S rRNA sequencing

**Fig. 1:**
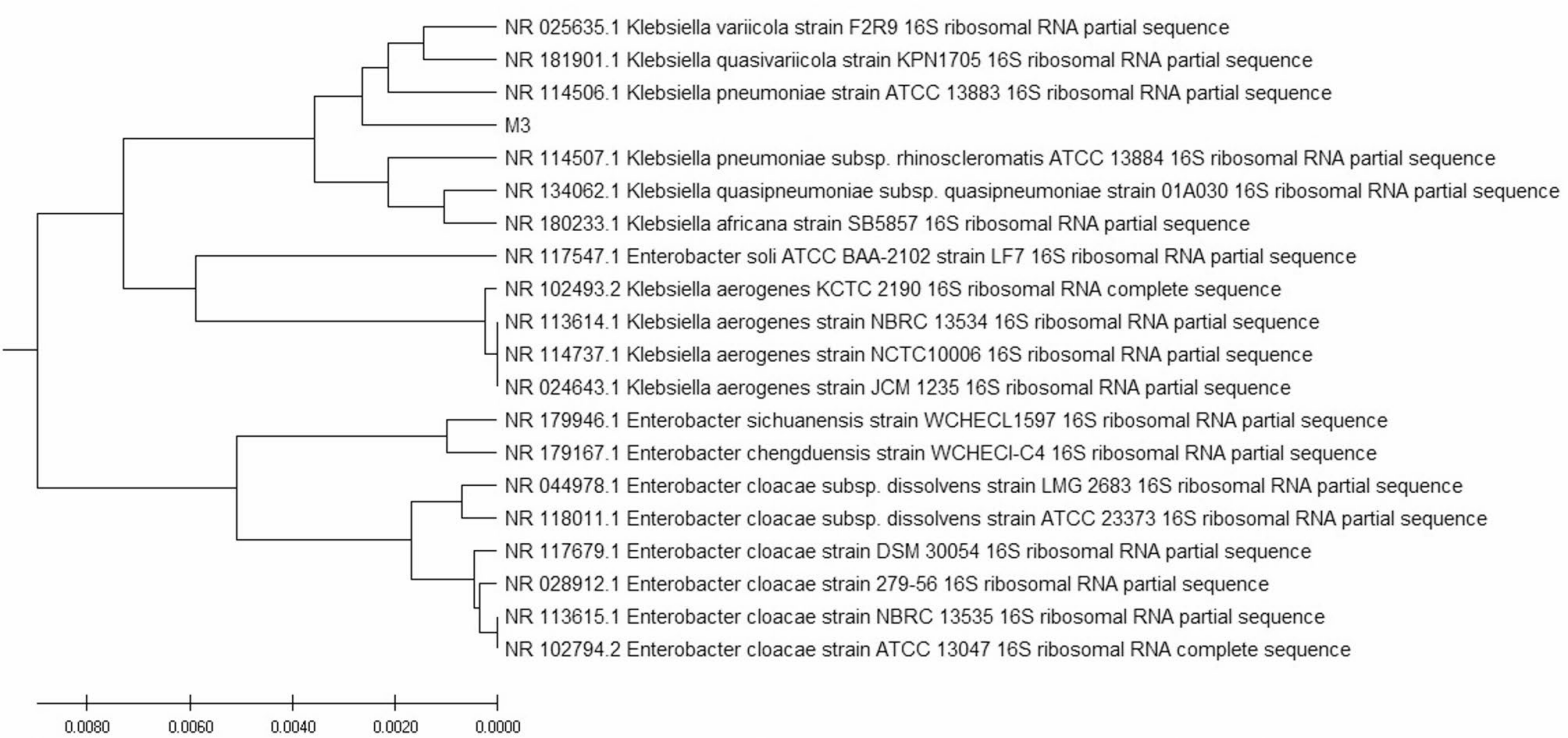
Phylogenetic tree showing sample 3 is *Klebsiella* sp. MK3 prepared using MEGA11 software (version 11).

After observing the peak production rate of around 67% demonstrated by isolate M3, we proceeded with further optimization. A 16s sequencing analysis was performed to characterize the bacterial isolate and generate a phylogenetic tree using the acquired sequences. The analysis of Blast results and the phylogenetic tree indicated a significant similarity between the M3 strain and three specific *Klebsiella* strains, as illustrated in Fig. 1. As a result, the taxonomic classification of the isolate was determined as *Klebsiella* sp. MK3. The same has been submitted to NCBI and accession number is PP109354.

### Qualitative and quantitative on wood waste supernatant

**Fig. 2:**
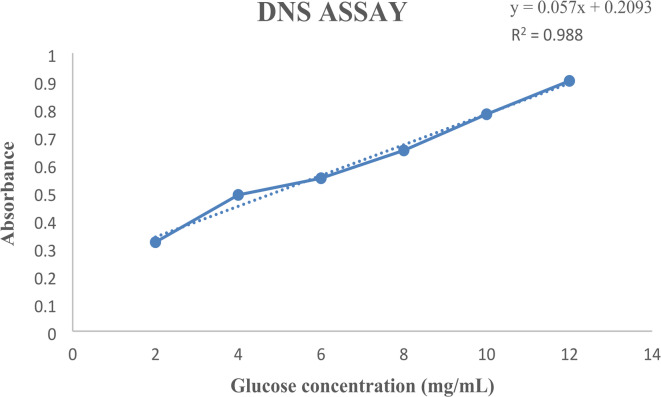
Standard graph of glucose using DNS test for reducing sugar.

The Molisch test confirmed the presence of carbohydrates through a purple ring. Absorbance values recorded for sal wood, teak wood, and their mixture were 0.722, 0.741, and 0.903, respectively, at 540 nm post one-third dilution for the mixture. Comparison with a standard DNS assay graph (Fig. 2) indicated that hydrolyzed sal and teak wood contained approximately 36 mg/mL of sugar^25^. During fermentation, the selected isolates demonstrated the ability to utilize sugars from the wood hydrolysate efficiently. The color change from purple to light brown after 24 h indicates active fermentation and successful substrate utilization^26^. The availability of diverse carbon sources can significantly impact microbial metabolism, influencing the yield and efficiency of fermentation. The observed color change suggests successful metabolic activity, but quantitative assessments, such as measuring biomass accumulation, pH changes, and gas production, would offer a more robust understanding of microbial growth dynamics.

### Plackett and Burman design (PBD)

Design Expert 12 software optimized PHB production using two phases: Plackett-Burman Design (PBD) and Response Surface Methodology (RSM). PBD assessed 11 factors across 12 runs for each of the 19 isolates, focusing on Carbon, Nitrogen, and other key parameters. Isolate no. 3 excelled, surpassing a 60% production rate. Experiments were conducted in triplicate to ensure accuracy, using specific concentrations of wastewater and wood waste substrate, with the significance of factors determined by a t-test^27^. Table 4 shows the dataruns of PBD and the experimental data of PHB yield.

**Table 4:** PBD provided data runs and experimental data of PHB yield per 10 mL.

Std.	Run	A: carbon %	B: Nitrogen %	C: Ferric citrate %	D: Temperature Celsius	E: pH	F: Inoculum size micro mL	G: MgSO_4_.7H_2_O %	H: Na_2_HPO_4_%	J: K_2_HPO_4_%	K: Incubation period Hours	L: Trace elements %	PHA mg/10mL
7	1	4	0.01	0.001	28	9	2	0.1	0.4	0.1	96	1	57
11	2	4	0.01	0.01	40	9	2	0.01	0.1	0.4	48	1	60.5
5	3	1	0.01	0.01	28	9	10	0.01	0.4	0.4	96	0.1	41
8	4	4	0.2	0.001	28	5	10	0.01	0.4	0.4	48	1	59.5
3	5	4	0.01	0.01	40	5	10	0.1	0.4	0.1	48	0.1	51
1	6	4	0.2	0.001	40	9	10	0.01	0.1	0.1	96	0.1	66
6	7	1	0.01	0.001	40	5	10	0.1	0.1	0.4	96	1	41
10	8	1	0.2	0.01	40	5	2	0.01	0.4	0.1	96	1	56.5
4	9	1	0.2	0.001	40	9	2	0.1	0.4	0.4	48	0.1	52.5
12	10	1	0.01	0.001	28	5	2	0.01	0.1	0.1	48	0.1	35.5
2	11	1	0.2	0.01	28	9	10	0.1	0.1	0.1	48	1	47
9	12	4	0.2	0.01	28	5	2	0.1	0.1	0.4	96	0.1	54.5

**Table 5:** ANOVA and fit statistics for the experimental design.

Model	3664.33	10	366.43	1099.30	0.0235	Significant
A-Carbon	1875.00	1	1875.00	5625.00	0.0085	
B-Nitrogen	833.33	1	833.33	2500.00	0.0127	
D-temperature	363.00	1	363.00	1089.00	0.0193	
E-pH	225.33	1	225.33	676.00	0.0245	
F-inoculum size	40.33	1	40.33	121.00	0.0577	
G-MgSO_4_.7H_2_O	85.33	1	85.33	256.00	0.0397	
H-Na_2_HPO_4_	56.33	1	56.33	169.00	0.0489	
J-K_2_HPO_4_	5.33	1	5.33	16.00	0.1560	
K-incubation period	33.33	1	33.33	100.00	0.0635	
L-trace elements	147.00	1	147.00	441.00	0.0303	
Residual	0.3333	1	0.3333			
Residual	1.33	1	1.33			
Cor Total	3532.00	11				
Std. Dev.	0.5774	R^2^	0.9999			
Mean	103.67	Adjusted R^2^	0.9990			
C.V. %	0.5569	Predicted R^2^	0.9869			

The presented data indicates a significant Model F-value of 1099.30 and a p-value of 0.0235, confirming the model’s effectiveness in explaining the variance in the response variable (Table 5). Key factors like A-Carbon, B-Nitrogen, and D-temperature significantly impact the system, evidenced by their low p-values and high F-values. Other factors like E-PH and G-MgSO_4_.7H_2_O also contribute significantly but less so than Carbon and Nitrogen. The Predicted R^2^ of 0.9869 closely aligns with the Adjusted R^2^ of 0.9990, suggesting robust model fit and predictive accuracy without overfitting. The model’s low Standard Deviation and Coefficient of Variation further confirm its precision, while an Adequate Precision of 110.353 indicates a reliable signal for navigating design space.

**Fig. 3:**
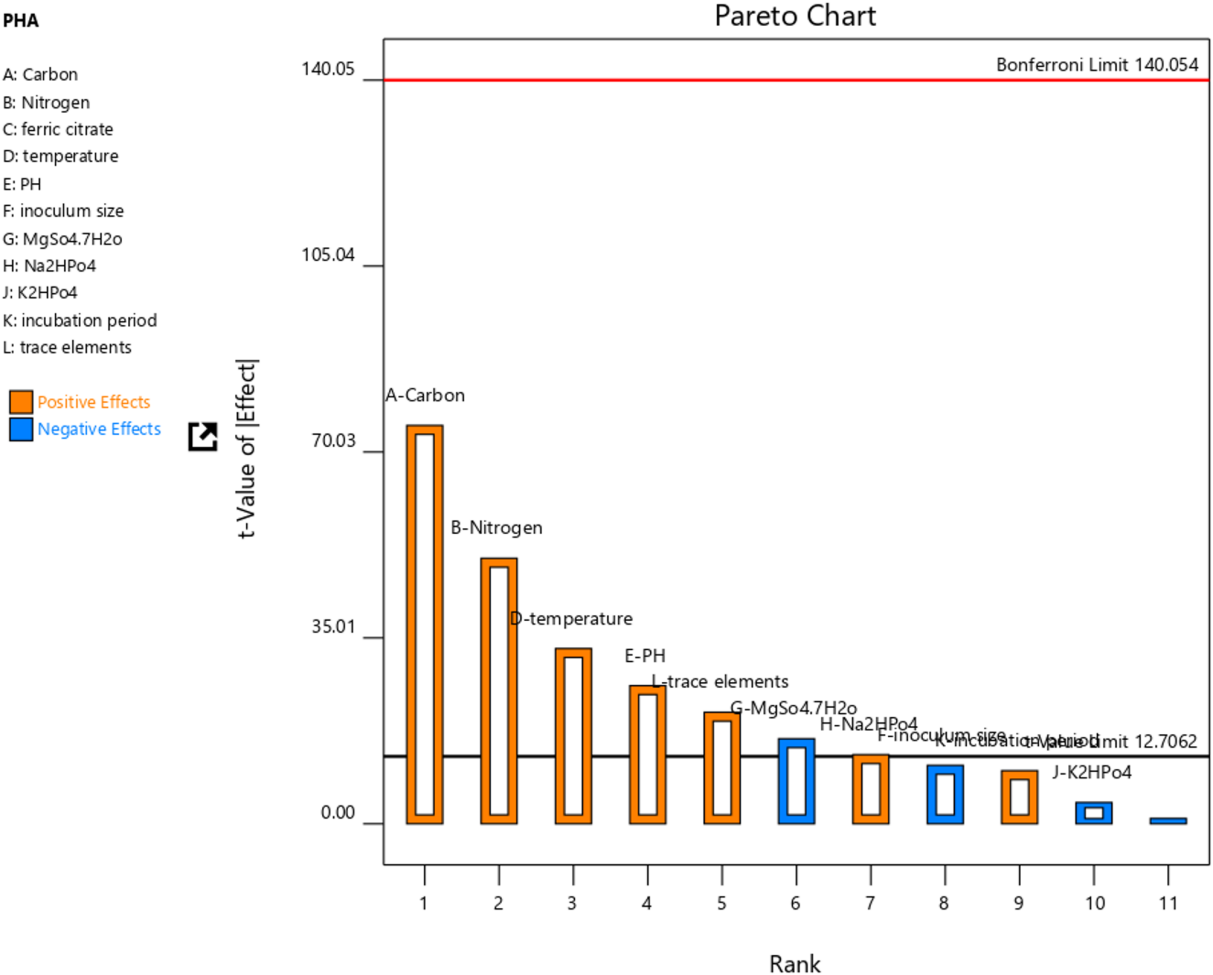
Pareto showing factors that are most affecting production rate.

A Pareto chart, depicted in Fig. 3, displays the relative significance of eleven factors in a Plackett-Burman design on PHB yield. Three factors (Carbon, Nitrogen, and Temperature) exceed the t-value limit of 12.70, indicating their substantial impact on the PHB response. The height of bars on the chart reflects each factor’s impact magnitude, allowing for easy comparisons. This chart is a valuable tool for identifying the most influential factors affecting the response variable and highlighting those needing further investigation or optimization. Additionally, it provides a concise and clear way to communicate the outcomes of the P.B. design^18^.

The analysis highlights A-Carbon as the most influential factor in the study, accounting for 51.16% of the effect with a substantial sum of squares at 1875. B-Nitrogen follows with a 22.74% contribution, while D-Temperature also plays a significant role at 9.91%. Other factors like E-PH, F-Inoculum Size, and G-MgSO_4_.7H_2_O have smaller impacts, contributing 6.15%, 1.10%, and 2.33% respectively. H-Na_2_HPO_4_ and L-Trace Elements also impact outcomes, contributing 1.54% and 4.01%. C-Ferric Citrate and J-K_2_HPO_4_ show minimal influence, suggesting their irrelevance in further analysis. This data assists in prioritizing factors for optimization and decision-making based on their contributions and effects^28^.

According to the results of the Pareto chart and numeric percentage contribution stats, carbon, nitrogen, and Temperature plays a vital role in PHB production yield. These three factors are considered for further optimization techniques using response surface methodology (RSM).

### Response surface methodology

Design Expert 12’s Response Surface Methodology (RSM) proves invaluable for result optimization, delving into intricate variable and response relationships through mathematical modeling. By predicting and optimizing response variables with a focus on interactive effects among factors, RSM aids in making informed decisions. Design Expert 12 facilitates this process with its user-friendly interface and statistical tools for experimental design, model fitting, response optimization, and result visualization. In this study, three optimization factors—Carbon, Nitrogen, and Temperature—are considered. The experimental and projected PHB values are presented in Table 6, demonstrating the efficiency of RSM in exploring the design space and determining optimal factor settings for achieving superior outcomes.

**Table 6:** Provides the experimental data of PHB using RSM and its experimental and predicted values.

Std.	Run	Factor 1 A: Carbon %	Factor 2 B: Nitrogen %	Factor 3 C: Temperature °C	Actual value PHA mg/20 mL	Predicted value PHA mg/20mL
5	1	1	0.105	28	77.00	75.75
11	2	2.5	0.01	40	90.00	90.25
17	3	2.5	0.105	34	112.00	112.80
7	4	1	0.105	40	85.00	85.25
8	5	4	0.105	40	98.00	99.25
14	6	2.5	0.105	34	113.00	112.80
2	7	4	0.01	34	78.00	76.50
1	8	1	0.01	34	74.00	73.50
13	9	2.5	0.105	34	113.00	112.80
6	10	4	0.105	28	100.00	99.75
3	11	1	0.2	34	73.00	74.50
9	12	2.5	0.01	28	80.00	81.75
10	13	2.5	0.2	28	103.00	102.75
16	14	2.5	0.105	34	113.00	112.80
4	15	4	0.2	34	109.00	109.50
12	16	2.5	0.2	40	105.00	103.25
15	17	2.5	0.105	34	103.00	110.80

#### Predicted vs. actual value

**Fig. 4:**
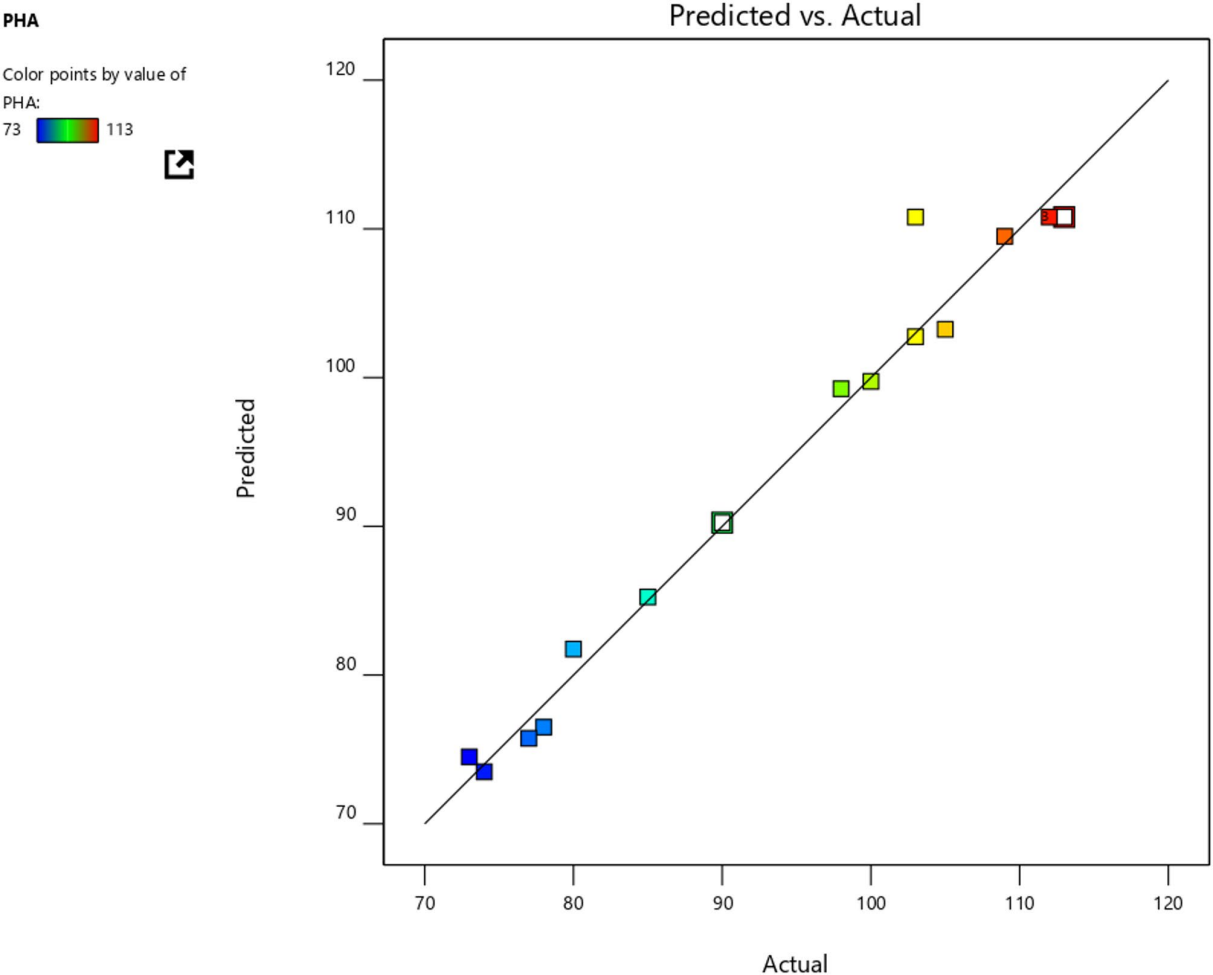
Showing predicted and actual value of PHB in graphical form.

The importance of the graph comparing predicted versus actual values lies in its capacity to assess the model’s goodness of fit. The model is deemed precise and dependable when the predicted values closely correspond to the actual values. Conversely, substantial deviations between the predicted and actual values suggest that the model might necessitate further refinement or investigation. As illustrated in Fig. 4, the data from 12 runs involving nine factors align along a straight line, indicating the accuracy of the data and the absence of outliers^29^.

The Model F-value of 29.89 confirms the statistical significance of the model, with significant terms including A, B, AB, A^2^, B^2^, and C^2^, indicating strong model terms. Non-significant terms can be pruned to enhance model efficiency. The Lack of Fit F-value at 0.25, with an 85.68% probability of being noise, suggests an excellent fit, as non-significant lack of fit is ideal Table 7). The Predicted R² of 0.9393 closely matches the Adjusted R² of 0.9909, underscoring model consistency. An Adequacy Precision of 34.659 indicates a robust signal, making this model highly reliable for navigating the design space^11,30^.

#### ANOVA for quadratic model

**Table 7:** ANOVA and fit statistics of quadratic model showing its significance.

Source	Sum of squares	df	Mean Square	F-value	*p*-value	
Model	3508.58	9	389.84	29.89	< 0.0001	Significant
A-carbon	722.00	1	722.00	55.36	0.0001	
B-nitrogen	578.00	1	578.00	44.32	0.0003	
C-temperature	40.50	1	40.50	3.11	0.1214	
AB	256.00	1	256.00	19.63	0.0030	
AC	25.00	1	25.00	1.92	0.2087	
BC	16.00	1	16.00	1.23	0.3046	
A^2^	1064.46	1	1064.46	81.61	< 0.0001	
B^2^	547.20	1	547.20	41.95	0.0003	
C^2^	101.09	1	101.09	7.75	0.0271	
Residual	91.30	7	13.04			
Lack of fit	14.50	3	4.83	0.2517	0.8568	Not significant
Pure error	76.80	4	19.20			
Cor total	3599.88	16				
Std. Dev.	1.48		R^2^	0.9960		
Mean	96.24		Adjusted R^2^	0.9909		
C.V. %	1.54		Predicted R^2^	0.9393	Adeq precision	34.6593
Source	Sum of squares	df	Mean square	F-value	p-value	

#### Final equation

**Y =** − 164.016 + 45.2164 * carbon + 333.684 * nitrogen + 10.6934 * temperature + 56.1404 * carbon * nitrogen + − 0.277778 * carbon * temperature + − 3.50877 * nitrogen * temperature + − 7.06667 * carbon^2 + -1263.16 * nitrogen^2 + − 0.136111 * temperature^2.

#### Model graphical representation

**Fig. 5:**
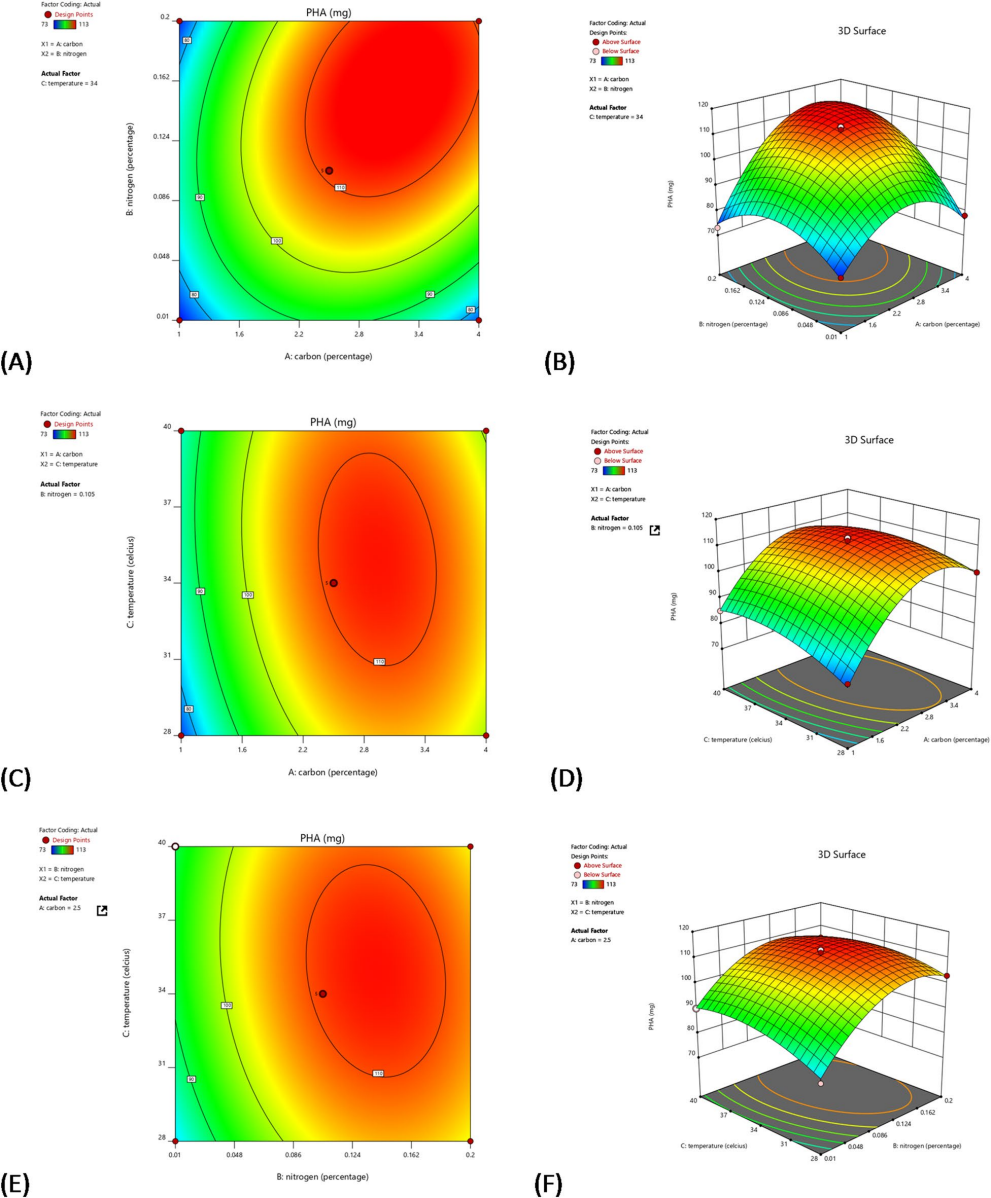
Illustrating contour plots and 3D-Surface response prepared using Design Expert (version 12.0) by StatEase: interactive effects of (A) and (B) varied the intensity of the carbon and nitrogen at temperature 34; (C) and (D) varied the intensity of the carbon and Temperature at nitrogen 0.105; (E) and (F) varied the intensity of the nitrogen and Temperature at carbon 1.7.

The contour and 3D surface plots (A–F), depicted in Fig. 5, utilize Response Surface Methodology (RSM) to explore the interactions between key factors—Carbon, Nitrogen, and Temperature—on PHA production. These visualizations effectively demonstrate how varying combinations of these factors influence PHA yield, providing insights into optimal operational conditions.

Plots A and B focus on the interaction between Carbon (X1) and Nitrogen (X2) percentages, with Temperature held constant at 34 °C. These plots reveal that the highest PHA production occurs at a Carbon level of approximately 2% and a Nitrogen level close to 0.1%. The red regions in both plots highlight areas of maximum PHA yield, suggesting that these are the most favorable conditions for PHA production within the tested range. Plots C and D examine the effects of Carbon percentage (X1) and Temperature (X2), keeping Nitrogen percentage fixed at 0.1%. The data shows that optimal PHA production is achieved when Carbon is around 2% and Temperature is near 37 °C. The contour plot (C) illustrates those moderate increases in both temperature and carbon percentage lead to higher PHA yields, indicated by the expanding red zone, which marks the region of peak production. Lastly, plots E and F analyze the interaction between Nitrogen percentage (X1) and Temperature (X2), with Carbon set at 2%. These plots indicate that increasing both Nitrogen and Temperature to their optimal values—approximately 0.1% for Nitrogen and 37 °C for Temperature—maximizes PHA yield. The 3D plot (F) vividly demonstrates this relationship, with a clear peak in the response surface at these conditions, confirming their effectiveness for enhancing PHA production.

#### Point prediction values

In the context of this study, the predicted values for carbon, nitrogen, and temperature are specified as 1.74, 0.105, and 37 °C, respectively, as outlined in Table 8. These values imply that maximizing PHB yield can be achieved under these specified conditions.

**Table 8:** Point predicted table for best PHB production.

Factor	Name	Level	Low level	High level	Std. dev.	Coding
A	Carbon	1.74	1.0000	4.00	0.0000	Actual
B	Nitrogen	0.1050	0.0100	0.2000	0.0000	Actual
C	Temperature	36.78	28.00	40.00	0.0000	Actual

#### Final production

Using Response Surface Methodology (RSM), the optimal conditions for the production of Polyhydroxybutyrate (PHB) by *Klebsiella pneumoniae* Mk1 were scaled down for a smaller volume. The ideal carbon concentration was determined to be 1.7% (17 g/L), nitrogen concentration 0.1% (1 g/L), and the incubation temperature was set at 37 °C. For a 500 mL substrate, the medium was prepared by mixing 236 mL of hydrolyzed wood waste water with 264 mL of distilled water. The hydrolyzed wood waste contained approximately 36 mg/mL of sugar content, determined by the DNS test standard graph. To this solution, 1 g of ammonium chloride was added as the nitrogen source.

**Fig. 6:**
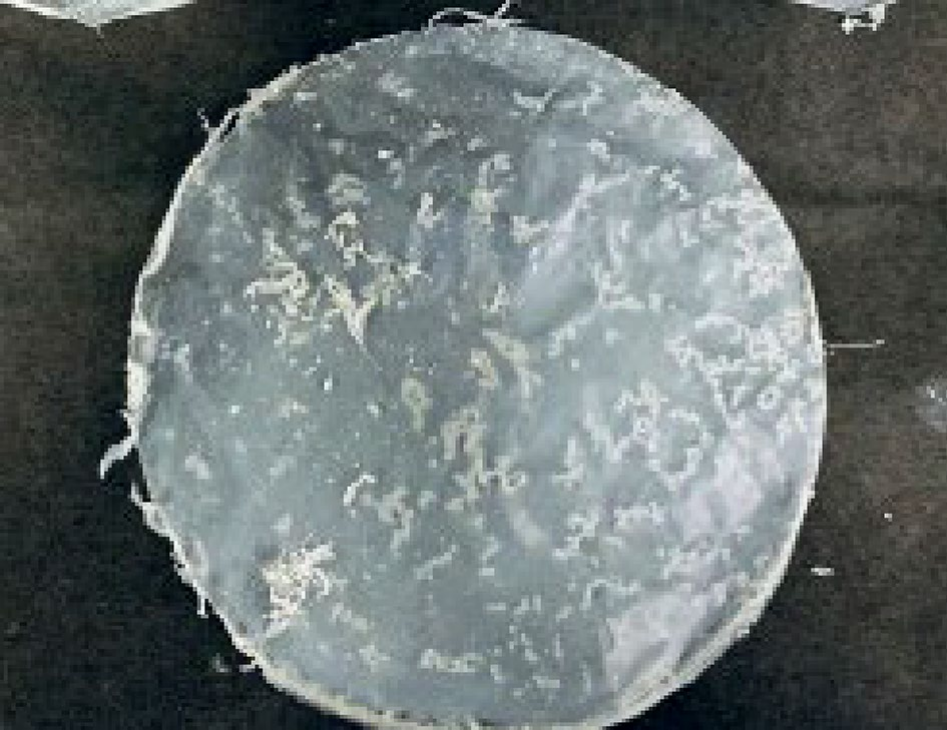
Displays the uniformly distributed form of the polyhydroxybutyrate (PHB) film following the evaporation of chloroform.

The medium was inoculated with the microbial isolate and incubated at 37 °C for 72 h. After the incubation period, the culture was centrifuged to separate the cells from the supernatant. The cells were then processed for PHB extraction. After adjusting for the smaller volume, the final yield of PHB (Fig. 6) obtained was approximately 2185.625 mg per 500 mL of the medium, equivalent to 4.37 mg/mL.

The availability and type of carbon source play a pivotal role in PHB production^24^. Pineapple peel, an inexpensive agro-waste, was successfully utilized as a carbon source for *Bacillus drentensis* BP17, yielding enhanced PHB accumulation through the optimization of pineapple peel concentration and fermentation conditions^31^. Similarly, Sachan et al. (2024) highlighted glucose as a key carbon source for *Bacillus paranthracis*, where its concentration was fine-tuned to achieve improved PHB synthesis^32^. For *Bacillus megaterium*, agricultural hydrolysates containing glucose and xylose were optimized for fermentation, demonstrating the impact of balanced carbon source utilization^33^. The nitrogen source and its concentration are critical for influencing microbial metabolism toward PHB accumulation. The C/N ratio was optimized in *Bacillus megaterium* PNCM 1890, revealing that a low nitrogen concentration promotes PHB^33^. Additionally, Kojuri et al. (2021) utilized ammonium sulfate as the primary nitrogen source and achieved enhanced PHB production through its optimized concentration in petrochemical wastewater^34^. pH and temperature optimization are fundamental for maintaining microbial activity and PHB yield. Trakunjae et al. (2021) reported that pH levels between 6.5 and 7.5 and temperatures of 30–35 °C were optimal for *Rhodococcus* sp. BSRT1-1, indicating that these factors significantly influence enzymatic activity and metabolic pathways^35^.

The global attention on sustainable and biodegradable polymers has spurred important advancement in the microbial synthesis of polyhydroxybutyrate (PHB) from lignocellulosic waste products. The present work shows that *Klebsiella* sp. MK3 effectively valorized an affordable substrate and presented a competitive yield relative to past literature by using sulfuric acid-treated wood waste to create 4.2 g/L of PHB. Under batch fermentation conditions, Abu-Thabit et al. (2022) investigated PHB generation from different lignocellulosic and plastic waste sources, noticing generally between 0.5 and 3.5 g/L, often requiring detoxification due to inhibitory molecules^36^. Attaining a yield of 4.2 g/L, the present work essentially avoids significant detoxification and shows the durability of *Klebsiella* sp. MK3 as well as the efficacy of 4% sulfuric acid pretreatment. Using *Paraburkholderia sacchari* to metabolize degradation monomers formed from PHB-based polymers, Zoghbi et al. (2023) produced PHB yields of about 2.3 g/L. Though different in approach, their reliance on bioplastic-derived carbon sources limits wider applicability in comparison to this work, which makes use of abundant, non-bioplastic wood waste^37^. With *Burkholderia ambifaria* E5-3, Arai et al. (2024) recorded PHB generation approaching 3.7 g/L using hydrolysates obtained from oil palm trunks. Although an inhibitor-tolerant strain was used, yields still fell short of those obtained in the current study^38^, thereby supporting once more the effectiveness of *Klebsiella* sp. MK3 and its substrate compatibility. Using acid-pretreated biomass, Saratale et al. (2022) developed a co-culture method producing PHB titers almost equal 3.5 g/L. Using the monoculture system of *Klebsiella* sp. MK3 used in this work avoids the complexity of co-culture systems that causes challenges in scaling and process control.

From ambient-alkaline-treated agricultural wastes^39^, Hossain et al. (2022) achieved PHB concentrations of 2.9 g/L; Davaritouchaee et al. (2023) reported just 1.1 g/L PHB from orange peel hydrolysates using a one-step oxidation process^40,41^.

This study highlighted the importance of maintaining substrate availability without causing inhibitory effects from substrate overload. The application of RSM has demonstrated significant potential in optimizing PHB production by systematically analyzing the effects of key parameters such as carbon and nitrogen sources, pH, temperature, and many more.

#### Qualitative analysis of PHB using UV-Vis spectroscopy

**Fig. 7:**
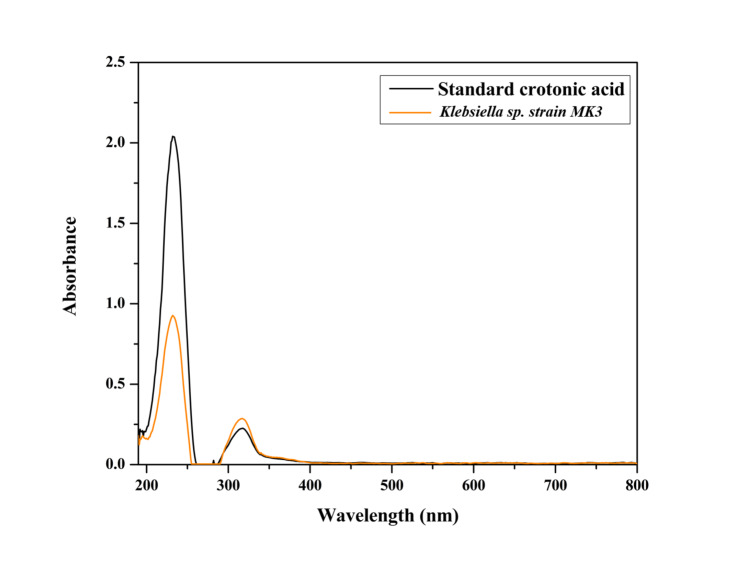
UV spectroscopy analysis of PHA (orange line) and crotonic acid (black line).

The UV spectroscopy result is significant in identifying and characterizing the presence of Polyhydroxybutyrate (PHB), a key type of PHA, by comparing it to the known crotonic acid standards^42^. Here are the key aspects of the result:

The similarity in peaks is crucial for confirming the presence of PHB, as crotonic acid is a known degradation product of PHB during polymer breakdown shown in Fig. 7.

The slight variations in peak intensities between the bacterial strains and the crotonic acid standard could be due to differences in the yield or purity of PHB extracted from the bacterial strain.

#### Fourier transform-infrared spectroscopy (FTIR)

**Fig. 8:**
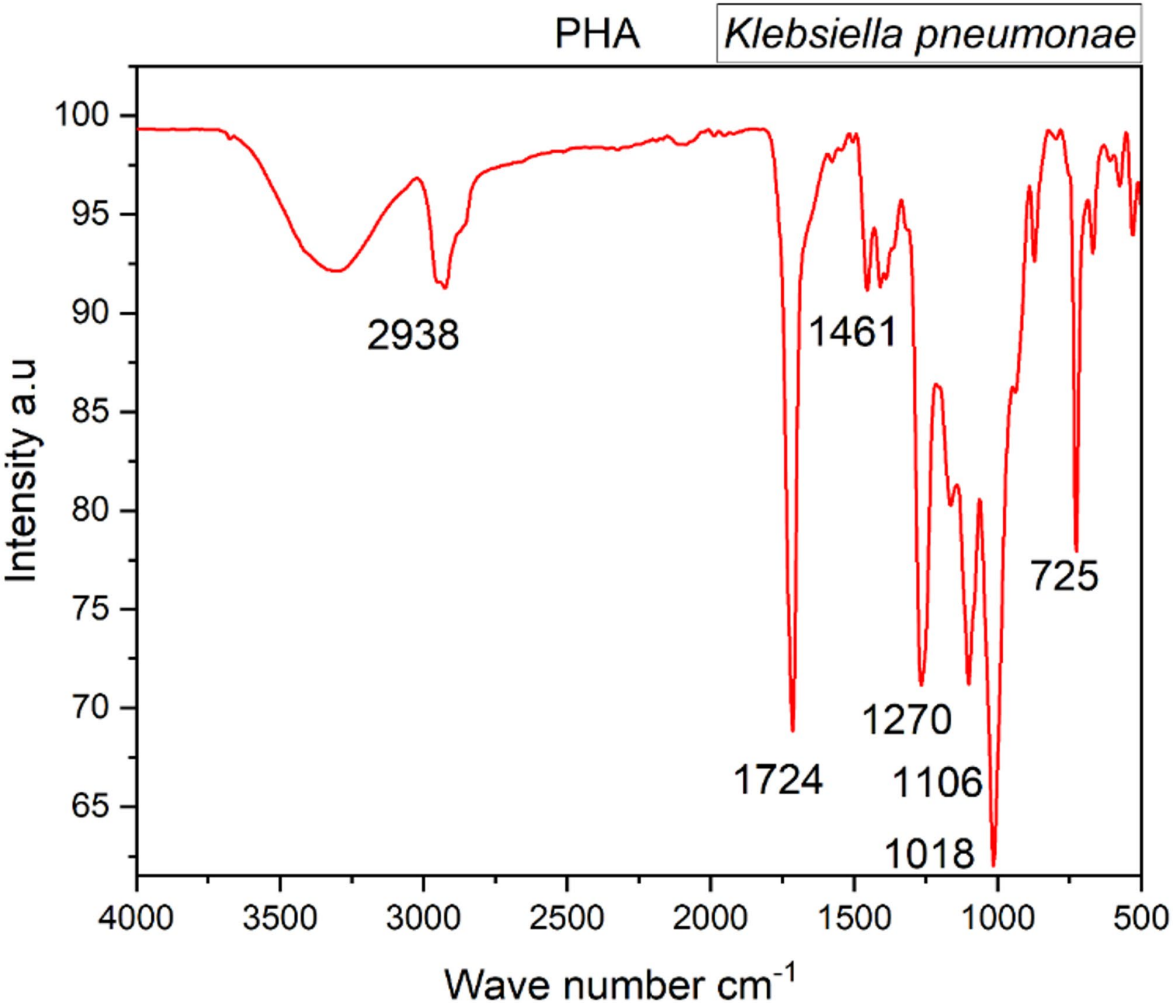
FTIR peaks representing the presence of PHB extracted from *Klebsiella* sp. MK3.

For PHB, the FTIR spectrum typically exhibits absorption peaks corresponding to the carbonyl (C=O) and ester (C-O) functional groups, which are distinctive features of the polymer backbone. These peaks offer insights into the polymer’s properties. In Fig. 8, specific absorption peaks include stretching of the OH. carboxyl group, C.H. bending, halogen compound, C=O ester, and CH, C-O, CH_3_, and OH groups of the polymer, occurring at wave numbers 725, 1018, 1106, 1270, 1461, 1724, and 2938 cm^–1^^43,44^. The analysis of these peaks provides crucial information about the chemical composition and structure of PHB. The presence of the ester group and methyl group indicates that the microbial bioprocessed polyester is a PHB polymer from the PHB family. PHB, within the PHB family, is distinguished by its unique chemical composition, featuring a methyl functional group (CH_3_) and an ester linkage group (-COOR). These specific chemical groups contribute to PHB’s thermoplastic nature, hydrophobic properties, high crystalline structure, and brittleness.

#### 1H and 13C NMR analysis

**Fig. 9:**
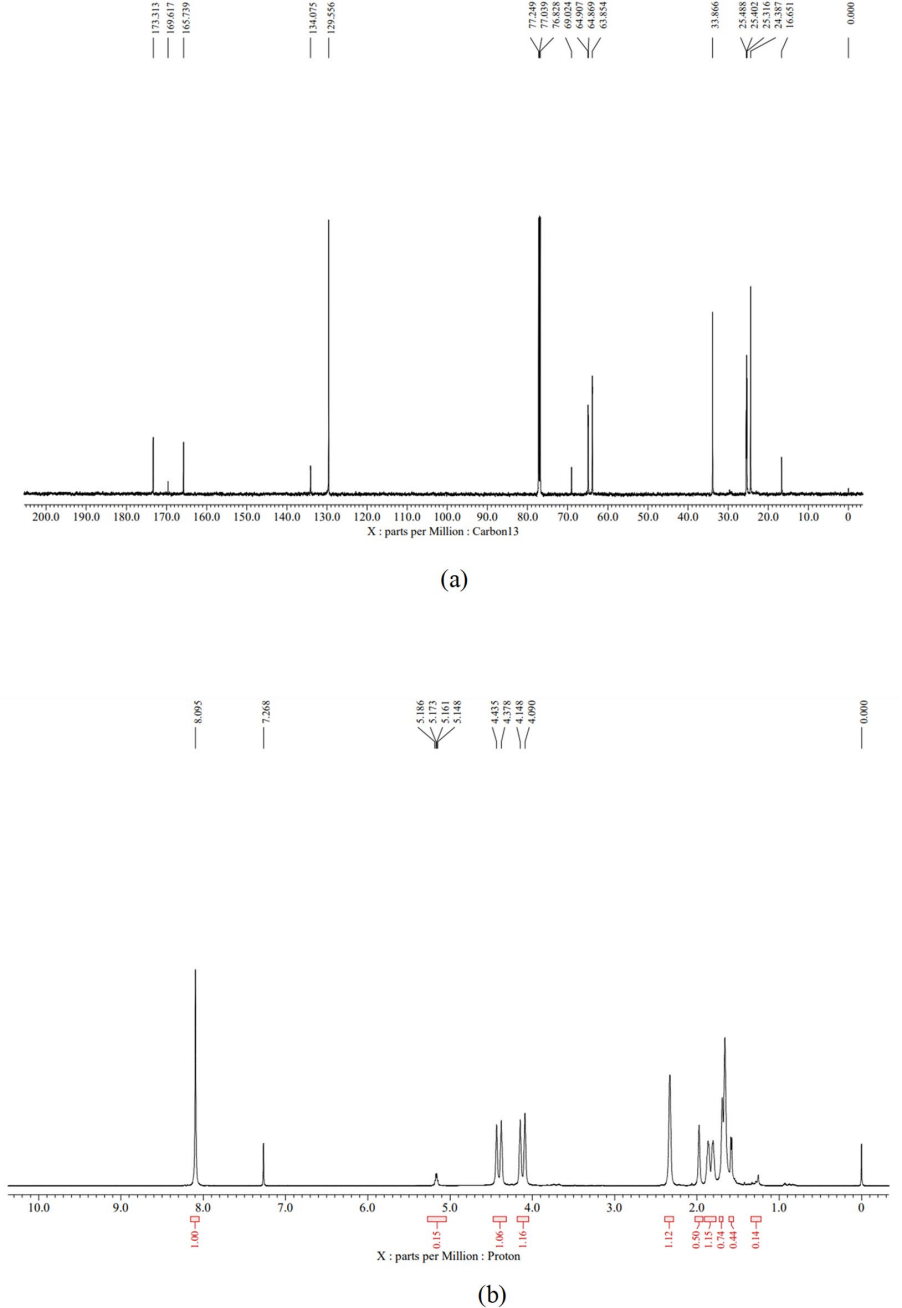
(a) 13C-NMR spectrum of the PHB extracted using chloroform. (b) 1H-NMR spectrum of the PHB extracted using chloroform.

The carbon-13 (C-13) NMR peaks as shown in Fig. 9a indicating various structural elements within the polymer. Peaks around 173.313, 169.617, and 165.739 ppm likely signify carbonyl (C=O) groups in the ester linkages of PHB’s backbone. Peaks at 134.075 and 129.556 ppm suggest aromatic or alkenyl carbons, potentially reflecting aromatic or unsaturated regions in PHB. Peaks around 77 ppm may represent carbons adjacent to ester groups. Peaks at 69 and 64 ppm could relate to the polymer backbone or side chains, while those below 33.866 ppm likely denote alkyl or alkyl halide groups from PHB’s side chains. In the proton nuclear magnetic resonance (1 H-NMR) spectrum, as Shown in Fig. 9b peaks at 8.095 and 7.268 ppm suggest aromatic or alkenyl hydrogen environments, while peaks between 5.186 and 4.090 ppm indicate hydrogen atoms near double bonds or ring structures, possibly from the polymer backbone or side groups. Peaks around 4 ppm likely correspond to methylene (CH_2_) groups, common in PHB’s polymer structure^44^.

## Conclusion

This study delves into the innovative use of wood waste and wastewater mixture as substrate media for the production of polyhydroxybutyrate (PHB) facilitated by *Klebsiella* sp. Worldwide, concerted efforts are underway to promote Polyhydroxybutyrate (PHB) accumulation as a sustainable alternative to conventional plastics. Researchers, industries, and policymakers collaborate to advance PHB production technologies, exploring its diverse applications. This collective endeavor aims to mitigate plastic pollution and foster a greener, more environmentally friendly future. This research offers promising benefits, including cost reduction and decreased dependence on petroleum-based plastics, aligning with environmental sustainability goals. It finds utility in packaging, agricultural mulch films, biomedical applications, disposable items like cutlery, textiles, and 3D printing. Its eco-friendly nature aids in reducing environmental pollution and dependence on non-renewable resources, offering sustainable solutions across diverse sectors. Leveraging statistical methods such as the Plackett-Burman design (PBD) and response surface methodology (RSM) has proven instrumental in optimizing key factors influencing PHB production. The PBD highlighted significant effects of temperature, carbon concentration, and nitrogen concentration on PHB production. Subsequently, RSM pinpointed the optimal conditions for maximal PHB production, revealing that a carbon concentration of 1.7%, nitrogen concentration of 0.105%, and a temperature of 37 °C resulted in a maximum production rate of 4.37 mg/mL using a substrate media recomposed of wood waste and wastewater that is approx. two-folds. However, furthermore, kinetic modeling of sugar uptake and metabolite production could help predict the efficiency of different carbon sources in promoting microbial growth. The UV-Vis spectroscopy, FTIR & NMR confirms the produced product is PHB. This optimized approach holds promise for substantial cost savings in PHB production, fostering a more sustainable and eco-friendly manufacturing process.

## Data Availability

The Klebsiella sp. isolate has been submitted to NCBI under the accession number PP109354, ensuring accessibility for further research and reference.
